# Smart Freeze-Dried Bigels for the Prevention of the Sexual Transmission of HIV by Accelerating the Vaginal Release of Tenofovir during Intercourse

**DOI:** 10.3390/pharmaceutics11050232

**Published:** 2019-05-13

**Authors:** Araceli Martín-Illana, Fernando Notario-Pérez, Raúl Cazorla-Luna, Roberto Ruiz-Caro, María Dolores Veiga

**Affiliations:** Departamento de Farmacia Galénica y Tecnología Alimentaria, Facultad de Farmacia, Universidad Complutense de Madrid, 28040-Madrid, Spain; aracelimartin@ucm.es (A.M.-I.); fnotar01@ucm.es (F.N.-P.); racazorl@ucm.es (R.C.-L.); rruizcar@ucm.es (R.R.-C.)

**Keywords:** vaginal microbicides, controlled release of Tenofovir, pectin, chitosan, HPMC, mucoadhesive freeze-dried bigels, pH-sensitive systems

## Abstract

Sub-Saharan African women are still at risk from the human immunodeficiency virus (HIV), and sex with men is the main route of transmission. Vaginal formulations containing antiretroviral drugs are promising tools to give women the power to protect themselves. The aim of this work was to obtain freeze-dried bigels containing pectin, chitosan, or hypromellose for the vaginal controlled release of Tenofovir, which is accelerated in the presence of semen. Nine batches of bigels were formulated using different proportions of these polymers in the hydrogel (1, 2, and 3% *w*/*w*). The bigels obtained were freeze-dried and then underwent hardness and deformability, mucoadhesion, swelling, and drug release tests, the last two in simulated vaginal fluid (SVF) and SVF/simulated seminal fluid (SSF) mixture. The formulation containing 3% pectin (fd3P) has the highest values for hardness, resistance to deformation, and good mucoadhesivity. Its swelling is conditioned by the pH of the medium, which is responsive to the controlled release of Tenofovir in SVF, with the fastest release in the SVF/SSF mixture. fd3P would be an interesting smart microbicidal system to allow faster release of Tenofovir in the presence of semen, and thus increase women’s ability to protect themselves from the sexual transmission of HIV.

## 1. Introduction

According to 2018 UNAIDS reports, there were about 5000 new infections by the human immunodeficiency virus (HIV) every day in 2017. Around 66% of these were in sub-Saharan Africa, where women continue to be disproportionately represented in these data, with 59% of new infections by HIV among adults in that year [1]. Sex with men remains the most significant threat to women in terms of their infection by this virus [2]. “One in three women worldwide has experienced physical or sexual violence” according to UNAIDS, and this latter factor has a direct bearing on their vulnerability to HIV infection [1]. Vaginal formulations are therefore important as they give women the power they need to protect themselves from the heterosexual transmission of HIV [3].

The administration of antiretroviral drugs as topical pre-exposure prophylaxis (PrEP) [4]—also known as “vaginal microbicides” in this case [5]—is a HIV prevention tool which limits the systemic toxicities associated with oral PrEP [4]. Tenofovir (TFV), a drug that acts by blocking HIV-1 replication, was the first antiretroviral drug that safely demonstrated PrEP and post-exposure prophylaxis against HIV sexual transmission in animal models [6]. Various vaginal dosage forms containing TFV have been studied for this purpose, including gels [7,8], tablets [9,10], films [11,12], and rings [13,14]. TFV-based microbicide vaginal formulations have already shown efficacy in HIV prevention in women. However, clinical trials point to low patient adherence as one of the main reasons for failure [15,16].

One proposal to solve this problem is to design dosage forms for sustained drug release [17]. This requires systems that remain in the vaginal area, which can be achieved by formulating mucoadhesive systems. The components that can confer mucoadhesion on a vaginal formulation include certain polymers that not only allow it to remain adhered to the vaginal mucosa but also allow controlled drug release as they swell when they come into contact with an aqueous medium [18] and become transformed into hydrogels [19]. Some of these polymers have been used to obtain mucoadhesive vaginal dosage forms for the controlled release of anti-HIV drugs. Among them it is worth highlighting chitosan—alone [20] or in combination with pectin [21]—cellulose derivatives [22], and Carbopol^®^ [23]. 

Other systems that can include these polymers are bigels [24,25], structured biphasic systems resulting from mixing an organogel (or oleogel) and a hydrogel [26]. These formulations have several advantages since they combine the characteristics of both gels, such as the cooling and moisturizing effect, good spreadability and ease of withdrawal—making them more acceptable to patients—provided by the hydrogel and the ability to cross the skin barrier offered by the organogel [25,27]. In addition to the synergy between both types of gel, other benefits include the possibility of including both hydrophilic and lipophilic drugs in the same system and the ease of preparation [28]. Some of the properties of bigels make them a good choice for transdermal drug delivery [29]. Although several studies on bigels through this administration route have appeared in recent years [30], there is still limited research on bigels for vaginal administration. Singh et al. first formulated bigels containing Carbopol^®^ for the treatment of bacterial vaginosis; however no mucoadhesion tests were included in this work, and controlled release profiles were obtained only for 12h [31]. We subsequently proposed bigels for the vaginal administration of TFV that were freeze-dried to obtain more mucoadhesive systems with greater control over the release of the drug than was already offered by the hydrophobic character of the organogel [32].

Another important factor to consider in the design of vaginal formulations is the different pH of the vaginal fluid and the semen, as this can influence their properties. As human vaginal pH is acidic (4–5) and seminal pH is higher (around 7.5), vaginal pH increases during intercourse [33]. This fact can be exploited with pH-sensitive systems so that the vaginal release of the drug is triggered due to the change in pH in the presence of semen. Hydrogels [34], microspheres [35] and nanoparticles [33] have been formulated as pH-sensitive vaginal dosage forms for the prevention of sexual transmission of HIV. These kinds of smart dosage forms can be achieved by adding pH-sensitive polymers. They must contain ionizable functional groups with an acidic or basic character which prompt the molecule to donate or accept hydrogen ions from the medium [36]. Pectin is a hydrophilic polysaccharide present in the cell wall of most plants. Its structure is based on a linear chain of poly-α-(1→4)-dextro-galacturonic acid in which the carboxyl groups can be esterified by a variable number of methyl groups [37,38]. These carboxylic groups make pectin a pH-sensitive polymer with an acidic character [36]. Chitosan is a polysaccharide composed of the copolymers glucosamine and *N*-acetylglucosamine and obtained by the N-deacetylation of chitin, a component present in the shell of crustaceans [39,40]. This polymer is also pH-sensitive due to the presence of amine groups, which confer a basic character [36]. HPMC is a semi-synthetic polymer whose structure consists of a cellulose backbone that contains methyl and hydroxyl groups esterifying its anhydroglucose units to a variable degree [41,42]. It is a non-ionic polymer, so the drug release that depends on it can therefore be expected not to respond to pH [43]; however its mucoadhesive properties and ability to control drug release make it a polymer of choice in the formulation of vaginal dosage forms. 

With this background, the aim of this research was to obtain freeze-dried bigels based on pectin, chitosan, or HPMC as a mucoadhesive polymer for the controlled release of TFV in the vaginal environment, which is accelerated in the presence of semen, for the purpose of protecting women against the sexual transmission of HIV. It was previously verified that none of the components included in these bigels were toxic at different concentrations in a lymphoblastoid cell line (MT-2) and a uterus-derived cell line (HEC-1A), with a CC_50_ higher than 1000 µg/mL [20,32,44]. 

## 2. Materials and Methods

### 2.1. Materials

Pectin (from apple; Lot: BCBK7271V) was provided by Sigma-Aldrich^®^ (St. Louis, MO, USA) and is a high methoxyl pectin (79.91 ± 1.66%, [21]). Chitosan (viscosity: 37 mPa·S; Lot: 0055790) was supplied by Guinama S.L.U. (La Pobla de Vallbona, Valencia, Spain). The N-deacetylation of this polymer was determined previously and is 54.73 ± 4.26% [21]. HPMC (Methocel^TM^ K100M Premium CR Hydroxypropyl; Lot: SB13012N31) was a kind gift from Colorcon Limited (Dartford, UK). Sesame oil (from *Sesamum indicum*; Lot: BCBN4676V), sorbitan monostearate (Span^®^60, Lot: MKBS8956V) and polysorbate 60 (Tween^®^60, Lot: MKBT3178V) were provided by Sigma-Aldrich^®^ (St. Louis, MO, USA). TFV (Lot: FT104801501) was purchased from Carbosynth Limited (Compton, UK).

Demineralized water, obtained by a Milli-Q^®^ system, was also used. Glacial acetic acid was acquired from PanReac (Castellar del Vallès, Barcelona, Spain). All other products were of analytical grade.

### 2.2. Preparation of Fresh Formulations

TFV-based biphasic systems comprising an organogel and a hydrogel were formulated. The organogel was formed by sesame oil and Span^®^60. The hydrogel was based on a polymer—pectin, chitosan or HPMC—and Tween^®^60 was included as surfactant (Table 1). The most promising hydrogel/organogel proportion was selected according to a previous research work [32]. Using three polymers in three different proportions to form the hydrogel, we obtained nine different batches. In the case of the C batches, the hydrogel was produced in a 1% *v*/*v* acetic acid aqueous solution, as chitosan does not jellify in pure water but in the presence of an acid. Specifically, Berger et al. stated that “solubilisation of chitosan in an acidic aqueous medium […] is the simplest way to prepare a chitosan hydrogel” [45]. The drug was included in the hydrogel of all the bigels, and an additional 3% *w*/*w* was assumed for the composition shown in Table 1. 

The organogel was prepared by dispersing Span^®^60 in sesame oil in a double boiler at a temperature of approximately 70 °C. The hydrogel was obtained by suspending TFV in water and subsequently adding the polymer. Once the system had gelled, Tween^®^60 was added to the hydrogel using heat and manual stirring to aid its incorporation. Once both the organogel and the hydrogel were formed from each batch, they were heated in a water bath and mixed when they reached the same temperature, and manually stirred until a homogeneous system was obtained.

### 2.3. Freeze-Drying the Fresh Systems to Obtain the Final Formulations

The resulting systems were freeze-dried, for several reasons. Key aspects for the ultimate purpose of these formulations were considered, such as mucoadhesiveness and control over the drug release. Woolfson et al. [46] demonstrated that mucoadhesive forces in gels were lower than those observed for the equivalent freeze-dried systems. This can be attributed to vaginal fluid penetrating into a structure that already contains water between its polymer chains, resulting in lower and progressively weakened mucoadhesion due to overhydration. In freeze-dried gels, water from the medium diffuses in the structure and the mucoadhesive polymer subsequently swells in a controlled way, thus ensuring the interaction between the polymer chains and the vaginal mucosa. More controlled drug-release profiles were also obtained from the freeze-dried gels than the fresh ones. The authors explained this behaviour as being due to the higher viscosity of the gels obtained from the reconstituted freeze-dried systems and the subsequent higher tortuosity for diffusing the drug [46]. Lyophilized formulations are also easier to handle and have increased stability compared to fresh systems [47].

For the freeze-drying process, fresh bigels were previously dosed in polyvinyl chloride (PVC) blisters with alveolus dimensions of 8 × 23 × 9 mm, with one gram of bigel placed in each hole. They were then lyophilized in a Lio-Labor^®^ freeze dryer (Telstar, Barcelona, Spain), attaining a freezing temperature of −45 °C, a sublimation temperature of −45 to 25 °C and a sublimation pressure of 4.54 × 10^−4^ atm inside the chamber [48].

### 2.4. Scanning Electron Microscopy (SEM)

The microstructure of the resulting freeze-dried bigels was observed with a field emission scanning electron microscope (JEOL JSM-6335F, Tokyo, Japan) at 20.0 kV. A sample of each batch was cut in two parts and one of these halves was fixed on the microscope sample holder exposing its internal structure, then coated using a gold sputter module in a high-vacuum system.

### 2.5. Hardness and Deformability Test

This test was done using an expanded method of one proposed in a previous work [32] to evaluate the mechanical properties of the freeze-dried bigels, which will determine their suitability for vaginal administration [49]. A TA.XT*plus* Texture Analyser (Stable Micro Systems, Surrey, UK) was used with a 5 kg load cell and a cylinder probe with a diameter of 20 mm. Half a lyophilizate was fixed to the texture analyser table with double-sided tape. The probe was placed at an initial height of 20 mm above the table. In a compression and cyclical mode, the probe descended at 1 mm/s and, after reaching a trigger force of 0.49 N, pressed into the dosage form to a depth of 1mm at 1 mm/s. The probe then returned to the starting height at the same speed. The force applied by the probe vs. time was measured. The maximum force in the first compression cycle was taken as an indicator of the hardness of the formulation and the deformability was determined from the maximum forces in the various cycles. This assay was done in triplicate for each batch. The hardness data were statistically analysed using Student’s *t*-test (considering *p* < 0.05 as significant). 

### 2.6. Mucoadhesion Test

This test was done to evaluate the mucoadhesion ability of the freeze-dried bigels according to a previously published method [21]. The TA.XT*plus* Texture Analyser (Stable Micro Systems, Surrey, UK) was used with a 5 kg load cell and cylinder probe with a 20 mm diameter. The dosage form was fixed to the probe with double-sided tape. A sample of bovine vaginal mucosa obtained from a local slaughterhouse was fixed to the bottom of a 5 cm-diameter Petri dish with ethylcyanoacrylate (Loctite^®^, Henkel Ibérica S.A., Barcelona, Spain). The dish was then placed on the texture analyser table. The mucosa remained moistened in simulated vaginal fluid (SVF; pH = 4.2) prepared according to Owen and Katz [50] until the start of the assay. In compression mode, the probe descended at 1 mm/s and, after reaching a trigger force of 0.05 N, pressed the dosage form against the mucosa with a force of 2 N for 30s at 0.1 mm/s. The probe then returned at a speed of 0.1 mm/s to a height that ensured the total detachment of the lyophilizate from the mucosa. The force vs. time was measured. The work required to detach each freeze-dried system from the mucosa was recorded. This assay was done in triplicate for each batch. The data were statistically analysed using Student’s *t*-test (considering *p* < 0.05 as significant). 

### 2.7. Swelling Test 

This test was done to analyse how the structure of the freeze-dried bigels could change in the presence of vaginal and seminal fluids and how this could determine the release of TFV. SVF and a mixture of SVF and simulated seminal fluid (SSF) were used—also based on the proposal of Owen and Katz [51]—in a proportion of 1:4 *v*/*v* [33]. According to the method described by Mamani et al. [52], each lyophilizate was initially weighed and fixed to a 13 cm-diameter stainless-steel disc with ethylcyanoacrylate (Loctite^®^). Each disc was then immersed in 100 mL of the corresponding medium in a beaker and placed in an oscillating water bath (P SELECTA^®^ UNITRONIC OR, JP SELECTA S.A., Barcelona, Spain) at 37 °C and 15 opm to simulate in vivo conditions. The discs were removed from the beakers at predetermined times and weighed using a precision balance (METTLER^®^ AT 200, Mettler-Toledo S.A.E., Barcelona, Spain) after eliminating the medium with a paper towel. This assay was done in triplicate for each batch. The swelling ratio was calculated according to Equation (1):

Swelling ratio (%) = (*L*_t_ − *L*_0_)/*L*_0_ × 100 (1)

where *L*_t_ refers to the weight of the lyophilizate at a preset time and *L*_0_ to the weight of the lyophilizate before coming into contact with the medium (dry).

### 2.8. Drug Release Test

One of the main aims of this research work was to obtain dosage forms whose controlled release of TFV in vaginal fluid is accelerated in the presence of semen, thus affording women greater protection against the virus during intercourse. To determine the ability of the developed systems to release TFV in this pH-dependent manner, a drug release test was done in both SVF and the SVF/SSF mixture (1:4 *v*/*v*). Each lyophilizate was introduced in a 100 mL borosilicate glass flask containing the corresponding medium in a volume of 80 mL (sink conditions). The bottles were immersed in an oscillating water bath (P SELECTA^®^ UNITRONIC OR) at 37 °C and 15 opm. At preset times, aliquots of 5 mL were removed from each bottle and the volume was replaced with clean medium. After filtering through a 0.45 µm Minisart^®^ filter (Sartorius A.G., Goettingen, Germany), the amount of TFV released into the medium was quantified by UV-visible spectroscopy at a wavelength of 261 nm in a JASCO V-730 spectrophotometer. This assay was done in triplicate for each batch.

The similarity factor *f*_2_ was used to compare the drug release profiles, and the data obtained in this assay were processed to determine whether they fitted Korsmeyer-Peppas and Higuchi kinetics [53] in order to understand the mechanisms responsible for releasing the drug from the formulations. Although there are several kinetic models, these two were selected because diffusion and the structural modification of the systems are the processes involved in releasing the drug from the freeze-dried bigels. 

#### 2.8.1. Korsmeyer-Peppas kinetic

In general, this model corresponds to Equation (2):

*M*_t_/*M*_∞_ = *a*·*t^n^* (2)

where *M*_t_/*M*_∞_ is the fraction of drug released according to the dose, *a* is a constant that depends on the structural and geometric characteristics of the dosage form, *t* is time, and *n* is the exponent indicating the mechanism responsible for the drug release. In this case, diffusion predominates when the value of *n* is less than or equal to 0.5; values of between 0.5 and 1.0 indicate an “anomalous transport” based on diffusion and the structural modification of the dosage form; *n* values equal to 1.0 (“transport case II”) and over 1.0 (“transport super-case II”) show drug release due only to structural changes in the formulation. 

#### 2.8.2. Higuchi kinetic

This kinetic can be summarised by Equation (3), known as the “simplified Higuchi model”:

*Q*_t_ = *K*_H_*t*^1/2^ (3)

where *Q*_t_ is the amount of drug released at time *t* and *K*_H_ represents the Higuchi dissolution constant. Based on this model, the drug is released to the medium by a diffusion process according to Fick’s first law, and proportionally to the square root of time.

The Akaike Information Criterion (AIC), which corresponds to Equation (4), was used to determine the kinetics that best fit the data obtained from the drug release tests:

AIC = *N* × ln(SSR) + 2 × *p* (4)

where *N* represents the number of experimental data, SSR is the sum of squared residuals and *p* is the number of parameters in the model. The lowest AIC value indicates the model with the best statistical fit to the drug release data. 

## 3. Results and Discussion

### 3.1. Preparing the Fresh Formulations

In the absence of the drug, the prepared hydrogels are transparent and brown if they contain pectin, yellow in the case of chitosan, and colourless if the polymer is HPMC. When TFV is added to these hydrogels they become translucent and white, as when Tween^®^60 is added to the hydrogel without the drug. The incorporation of Span^®^60 to sesame oil at 70 °C produces a transparent yellow dispersion that becomes an opaque yellow/white semisolid at room temperature, thus forming the organogel. The ability of sorbitan monostearate to jellify oils was noted by Murdan et al. [54]. The bigels were obtained interposing the corresponding hydrogels and organogels; the batch containing 3% HPMC (3H) did not produce a homogeneous bigel and so was excluded from further evaluation. All the resulting bigels have a homogeneous bright creamy white appearance, slightly brownish in the case of P batches and slightly pinkish in C batches. 

### 3.2. Freeze-Drying the Fresh Systems to Obtain the Final Formulations

**Figure 1:**
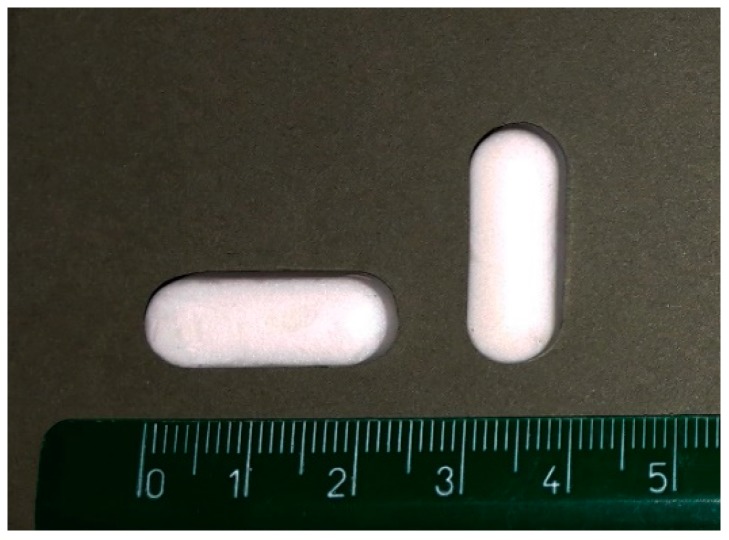
Appearance and dimensions of the freeze-dried bigels.

All the freeze-dried bigels (fd bigels) are white in colour and soft to the touch and have the appearance and dimensions shown in Figure 1. They all exhibit sufficient flexibility to allow them to be extracted from the blisters and handled without breaking, except for the bigels in batches 1P (fd1P) and 1C (fd1C). This indicates a poor mechanical stability, so these two systems were excluded from further evaluation. 

### 3.3. Scanning Electron Microscopy (SEM)

**Figure 2:**
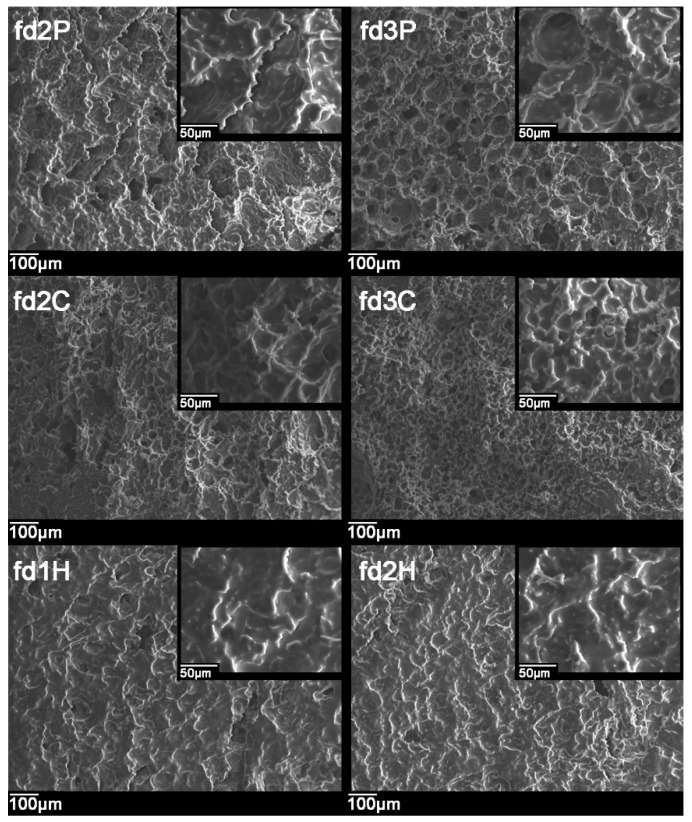
Cross-sectional micrographs of the freeze-dried systems obtained by scanning electron microscopy (SEM) at 100 and 500 times magnification.

Images obtained by SEM of the cross sections of the freeze-dried bigels are shown in Figure 2. A porous structure can be seen in all cases as a result of the sublimation of the frozen water in the freeze-drying process [55]. However, some differences can be established between the different batches. Since the batches differ in the concentration and/or nature of the polymer included in the hydrogel in the systems, these are the factors that will explain the differences. 

Depending on the polymer concentration, smaller pores can be seen in bigels with a higher amount of polymer (fd3P << fd2P, fd3C < fd2C, fd2H < fd1H); this phenomenon was also reported by authors such as Shen et al. and Furst et al. [56,57]. A higher concentration of polymer can be considered to produce greater viscosity and a denser polymeric framework in the corresponding hydrogel, resulting in smaller water droplets being trapped inside, whose elimination during the freeze-drying process gives rise to the corresponding pores [58]. The arrangement of the polymer chains during the formation of the hydrogel can be said to vary with the amount of polymer; this is more obvious in the case of pectin batches. 

When comparing systems that contain different polymers but in the same proportions, freeze-dried bigels containing pectin can be seen to have larger pores than bigels based on chitosan (fd3P > fd3C and fd2P > fd2C). Batches containing HPMC reveal a microstructure with less defined pores that appear to be connected, forming ducts.

### 3.4. Hardness and Deformability Test

**Figure 3:**
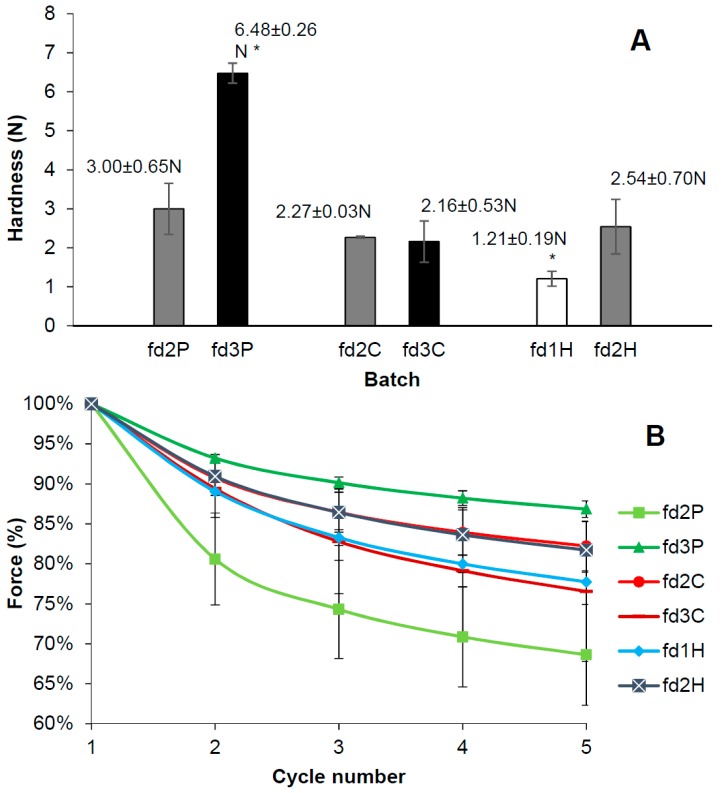
Values for hardness (**A**) and deformability curves (**B**) for each freeze-dried batch. Asterisks in Figure 3A indicate batches with statistically different values from the rest.

The hardness and deformability results of the freeze-dried systems are shown in Figure 3A,B, respectively. As can be seen in Figure 3A, the proportion of pectin and HPMC in the freeze-dried bigels affects their hardness, and the greater the proportion of the polymer, the higher the value of this parameter. Student’s *t*-test corroborated these differences. This direct correlation between hardness and polymer concentration was also found by Furst et al. for hydroxyethyl cellulose sponges [49]. A porous structure resulting from the freeze-drying of a hydrogel could be expected to be harder due to the greater density of the three-dimensional network of the previous hydrogel. The previous SEM micrographs suggest that smaller pore size can be associated with the greater hardness of freeze-dried bigels containing pectin or HPMC. However, this does not occur in the case of chitosan as there is no difference between the hardness of fd2C and fd3C. Student’s *t*-test showed that the nature of the polymer at the same concentration (2%) has no effect on hardness, since no significant differences were found between fd2P, fd2C, and fd2H. However, this test also confirmed that fd3P is significantly harder and fd1H significantly less hard than the other batches. 

Figure 3B shows the deformability curves obtained with the maximum force applied in each compression cycle. Force values were expressed as a percentage, considering the maximum force of the first cycle to be 100%. In all cases the force required to compress the systems by 1 mm decreases from one cycle to another, indicating that these freeze-dried bigels are increasingly deformable. This suggests that the deformability of batches based on pectin or HPMC becomes greater as the proportion of polymer in the hydrogel (fd2P >> fd3P and fd1H > fd2H) decreases, due to a more pronounced reduction in force. It is especially significant in the case of pectin. Furst et al. also obtained a reverse correlation between polymer proportion and the deformability of hydroxyethyl cellulose sponges [49]. In view of the results of the previous tests, deformability increases with larger pore sizes and the hardness of the structure resulting from lyophilisation decreases. In batches based on chitosan, the higher the concentration of polymer (fd3C > fd2C), the more deformable the system, although with no significant differences between them. These results also reveal that the proportion of polymer affects the deformability in different ways depending on the nature of the polymer included in the hydrogel. 

According to the results of these tests, batch fd3P is the hardest and least deformable freeze-dried bigel of all the formulated batches, making it the most suitable for vaginal administration. 

### 3.5. Mucoadhesion Test

The phenomenon of mucoadhesion is based on establishing interfacial forces between a material—the dosage form in this case—and a mucous membrane, whose surface is upholstered by a mucus layer [59]. The main components of this mucus layer are water and mucin glycoproteins, which possess sialic and sulphated residues. These groups are ionized at a pH of over 2.6 (pKa), giving a negative charge to the molecule [60,61]. The mucoadhesion of pectin is usually attributed to hydrogen bonds between its carboxylic groups and mucin glycoproteins [60]. However, the pKa of this polymer (3–4) is closer to vaginal pH, so some of its carboxylic groups could be ionized in this medium. This would create an electrostatic repulsion between the carboxylic groups and the similarly negatively charged mucin glycoprotein groups. Sriamornsak et al. suggested this repulsion could aid the formation of bonds by polymer coil expansion. In pectins with a high degree of esterification, methoxyl groups confer a hydrophobic character on the polymer, resulting in greater adsorption on the mucin surface [61]. Chitosan amino groups are positively ionized at pH values of less than 6.2–7 (pKa) [62,63], so they interact ionically with the negatively charged residues of mucin glycoproteins and produce the mucoadhesion of chitosan. Hydrogen bonds between the hydroxyl and amino groups of chitosan and mucus also contribute to the mucoadhesion of this polymer. Since HPMC is a non-ionic polymer, the pH of the medium does not affect its mucoadhesion, which is explained by the formation of bonds (including hydrogen bonds) between its hydroxyl groups and the functional groups of the mucus components [64].

**Figure 4:**
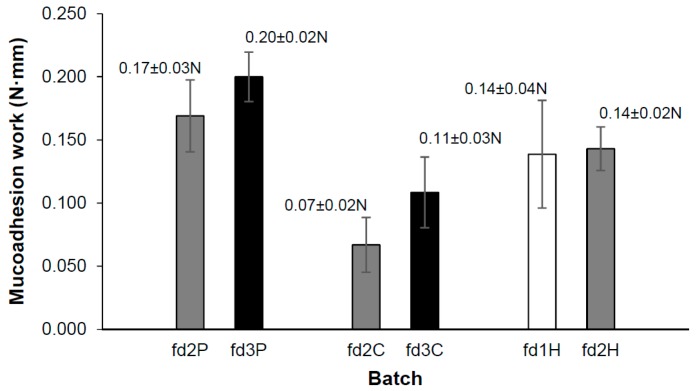
Average mucoadhesion work values for the freeze-dried batches.

Figure 4 shows the average values of the work required to detach each freeze-dried system from the mucosa, a parameter that we will call “mucoadhesion work”. As can be observed, this parameter is modified in the same way by varying the proportion of the polymer, whatever its nature, and therefore increases with the proportion of pectin, chitosan, or HPMC in the hydrogel, although only slightly in the last case. This expected correlation between the polymer concentration and the mucoadhesion work, which was also indicated by Furst et al. [57], may be because greater amounts of polymer allow more interactions with mucus per unit of surface area. However, Student’s *t*-test failed to establish any significant differences between the mucoadhesion work data for the batches containing different proportions of the same polymer. The statistical analysis revealed no significant differences between fd2P and fd2H, but significantly higher values of these batches with respect to fd2C. 

In view of the above, it can be confirmed that these variations in the proportion of polymer do not affect the mucoadhesion work of the systems, although the nature of the polymer (at the same concentration) does. Thus freeze-dried bigels containing pectin and HPMC have better mucoadhesive properties than those based on chitosan.

### 3.6. Swelling Test 

**Figure 5:**
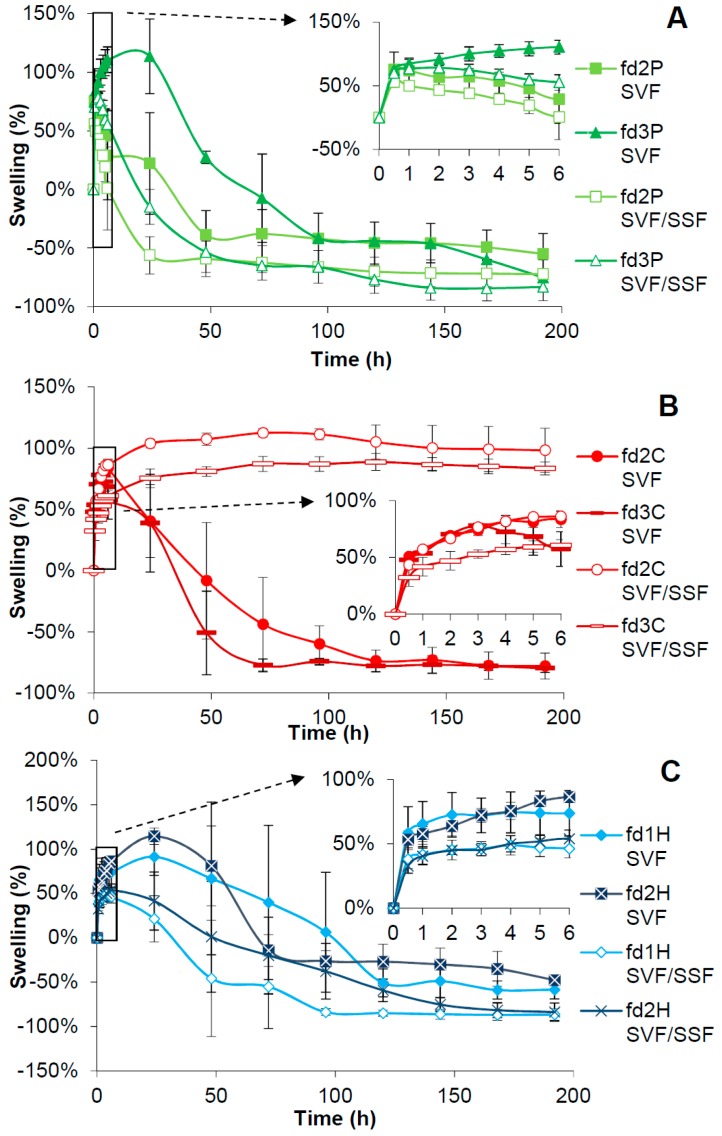
Swelling profiles obtained from tests in simulated vaginal fluid (SVF) and SVF/simulated seminal fluid (SSF) mixture for freeze-dried bigels containing pectin (**A**), chitosan (**B**), and HPMC (**C**). In each graph the first 6 h of test are amplified at the top right.

Figure 5 shows the data resulting from the swelling test of the freeze-dried systems in both SVF and the SVF/SSF mixture. Each graph groups the swelling profiles of the batches containing the same polymer in both media. In most cases, an initial swelling increase can be observed until a maximum value is reached, as the dominant process in this first stage is water capture from the medium. The mucoadhesive polymer traps the water in the three-dimensional structure of each freeze-dried bigel, resulting in the formation of a gel [65]. This process can therefore be considered to reconstitute the fresh bigels. It should be noted that these formulations do not swell excessively, unlike other vaginal dosage forms such as tablets [66,67], which makes them more comfortable for patients. This reduced swelling of bigels can be attributed to the small amount of swellable polymer in the dosage form and to the hydrophobic character conferred by the organogel. After the aforementioned maximum swelling value, the weight of the systems progressively decreases as they become destructured by erosion and/or dissolution. 

As can be seen in Figure 5A, the batches containing pectin begin to gain weight in the same way in SVF. However, fd2P reaches its maximum swelling value faster than fd3P (0.5 h as opposed to 24 h). fd2P begins to lose its structure earlier, and has significantly lower swelling values than fd3P from 3 h to 48 h. After this point, both batches show similar profiles until the end of the assay. The swelling profiles are more similar in the SVF/SSF mixture, as fd2P presents its maximum weight at 0.5 h and fd3P at 2 h. Nevertheless, in this short period the weight of fd2P diminishes faster than fd3P, with significant differences from the beginning of the test to 24 h. From 48 h on, these differences disappear until the end of the test, as fd2P and fd3P have similar weight variations. The fact that the lower the proportion of pectin, the sooner the maximum swelling value is reached could be because a lower amount of polymer requires less time to rehydrate [57]. Regarding the swelling of the same batch in both media, fd2P attains its maximum weight at 0.5 h in both SVF and the SVF/SSF mixture. Both profiles are similar, although a significant difference can be observed at 24 h, when this batch has a higher value in SVF than in the SVF/SSF mixture. More differences are observed for fd3P. This batch continues swelling for longer in SVF (until 24 h), so its weight loss begins later than in the presence of SSF; this freeze-dried bigel shows significantly higher swelling values in SVF than in the SVF/SSF mixture from almost the start of the test. This faster loss of structure in the SVF/SSF mixture than in SVF is due to the acidic character of pectin. This polymer is acid-stable [68] while highly soluble at a pH equal to or greater than 7 [69]. At the pH of the SVF/SSF mixture (around 7.5), the carboxylic groups of the polymer are ionized and generate a mutual repulsion that hinders the association between the polymer chains and prevents the formation of a gel. However, at vaginal pH these functional groups can be considered non-ionized, which allows the cross-linking of the polymer chains and results in a gel [70].

Figure 5B shows the swelling profiles of freeze-dried bigels containing chitosan. In SVF they have practically overlapping profiles until 3 h, when fd3C reaches its maximum swelling. Batch fd2C does so at 6 h, so its weight decreases later and less markedly, but with significantly higher values than fd3C at only 6h into the test. In the SVF/SSF mixture, the two stages of the swelling profiles mentioned above are not observed in the case of chitosan-based batches. Initially, fd2C and fd3C undergo a weight increase until they reach a value that remains almost constant until the end of the test. Although fd2C and fd3C exhibit the same behaviour in this medium, there are differences between their swelling degrees; fd2C has higher values than fd3C, which are significant from 1h to 96 h. Their maximum weights are reached at 72 h in the case of fd2C and at 120 h for fd3C, both in the peculiar swelling plateaus that characterize these batches. In this case, the profiles obtained in the SVF/SSF mixture also reveal a relationship between the lower polymer concentration and the shorter time taken to reach maximum swelling. Very different swelling profiles are obtained in both media for batches containing chitosan. For fd2C, the values recorded in SVF and the SVF/SSF mixture overlap until 6 h. From this point on, the weight decreases progressively after this batch reaches its maximum value in SVF, with values that are very significantly lower than in the SVF/SSF mixture. In the case of fd3C, lower swelling values were obtained in the SVF/SSF mixture at the start of the assay, and were significant until 3 h. However, from 6 h on, fd3C shows the same behaviour as fd2C, although significant differences can be seen between both media from 48 h. These differences in the swelling profiles of chitosan-based batches from one medium to another can be explained by the basic character of this polymer. At vaginal pH, its amino groups are protonated, causing the chitosan to dissolve in the medium. However, at a higher pH—as in the SVF/SSF mixture—these functional groups are non-ionized, so the polymer becomes insoluble and forms a precipitate [62,71].

The weight evolution of freeze-dried batches containing HPMC in both media can be seen in Figure 5C. fd1H and fd2H have very similar profiles in SVF and reach their maximum weights at 24 h. Although fd1H appears to maintain its structure longer and lose weight more slowly than fd2H, their values are not significantly different at any point in the test. The swelling profiles of these batches are also similar in the SVF/SSF mixture, although there are some differences between them. While fd1H reaches its maximum at 4 h, fd2H does so at 6 h, so fd1H begins to lose its structure earlier, with lower values than fd2H that are significant at 96h and 120h. Again it is worth noting the correlation between the greater proportion of polymer and the longer the time taken to reach the maximum swelling value. Both fd1H and fd2H swell more in SVF than in the SVF/SSF mixture, and this difference is significant for both batches during much of the test. However, it is more evident for fd1H, since fd2H has very similar swelling values in both media at 72 h and 96 h due to its very acute weight loss from 48 h to 72 h. This could be because the nature of the medium determines the arrangement of the polymer chains and the solid–liquid interaction in the reconstitution of the hydrogel. Tritt-Goc et al. reported that the diffusion mechanism of a solvent into HPMC matrices varies depending on its pH and related it to the chemical exchange between the medium and the polymer [43].

### 3.7. Drug Release Test

**Figure 6:**
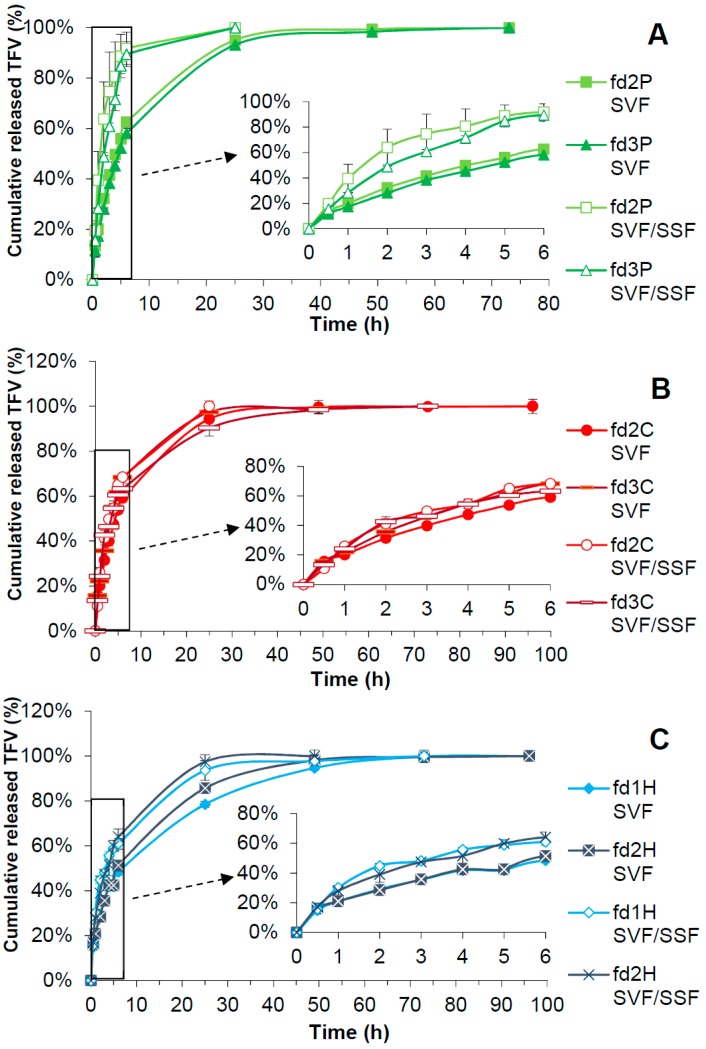
Cumulative drug release profiles obtained from tests in SVF and in SVF/SSF mixture for freeze-dried systems containing pectin (**A**), chitosan (**B**), and HPMC (**C**). In each graph the first 6 h of test are amplified at the bottom right.

Figure 6 shows the results obtained from the drug release tests of the freeze-dried bigels in both SVF and the SVF/SSF mixture. Each graph groups the release profiles of the batches containing the same polymer in both media. 

According to Figure 6A, batches fd2P and fd3P release the total amount of TFV in 72 h in SVF, although over 90% of TFV is released by 24 h. Both batches show almost the same drug release profile in this medium, maybe slightly more controlled for fd3P. According to the *f*_2_ similarity factor, they can be considered equivalent. Relatively similar drug release profiles were also obtained for fd2P and fd3P in the SVF/SSF mixture, in which both batches delivered TFV for 24 h. However, *f*_2_ did not reveal any similarity between these two profiles in this case, which could be due to the fact that the average values of TFV released from fd2P are higher—and therefore less sustained—than those of fd3P from 1 h to 4 h of the test. The faster loss of structure of fd2P observed in the swelling profiles would explain these minor differences between the release profiles of fd2P and fd3P. Notable differences can be observed when comparing the profiles in both media. The release of TFV is faster in the SVF/SSF mixture than in SVF, with significant differences from the first hour of the assay for both fd2P and fd3P, which were supported by the *f*_2_ factor. In the SVF/SSF mixture, around 90% of the drug is released in the first 6h of the test, whereas only about 60% of the dose is released from the same formulations in SVF. This agrees with the results obtained from the swelling test, which showed a faster loss of structure in the SVF/SSF mixture than in SVF, since pectin is more soluble at a higher pH. 

Figure 6B shows the drug-release profiles of batches containing chitosan. In SVF, fd2C and fd3C release the drug for 96 h and 72 h respectively, although over 90% of the dose is released at 24 h in both cases. Higher values of TFV released from fd3C than fd2C can be observed from 3 h to 6 h. Despite this, the *f*_2_ similarity factor did not indicate any significant differences between these profiles. In the SVF/SSF mixture, fd2C and fd3C allowed a controlled release of TFV for 24 h and 72h respectively, although around 90% of the drug is delivered in 24 h in the second case. Differences can be noted at 24 h, when fd2C reaches a higher percentage of released drug than fd3C; however, their profiles are equivalent based on the *f*_2_ value. Although some differences can be observed when comparing the release of TFV in SVF and in the SVF/SSF mixture, the *f*_2_ factor proved they are significant neither for fd2C or fd3C. Considering that freeze-dried bigels containing chitosan maintain their structure in the SVF/SSF mixture, a more sustained release of TFV could be expected from C batches in this medium than in SVF, and yet there are no significant differences between the release profiles of both media. This could be because chitosan precipitates at the pH of the SVF/SSF mixture, thus becoming a solid additive which is unable to control the release of the drug. 

As can be seen in Figure 6C, a controlled release of TFV for 96 h was obtained in SVF for batches containing HPMC, although the release from fd1H should only be considered until 72 h, since the very low increase in the percentage of drug released in the last 24 h would not be effective according to Karim et al. [72]. Over 90% of the drug is released at 48 h for both batches. The *f*_2_ value indicates that the drug release profiles of fd1H and fd2H are similar, although fd2H shows significantly higher TFV release values than fd1H at 24 and 48 h. In the SVF/SSF mixture, fd1H releases the drug for 72 h while fd2H does so for 48 h, although both batches exceed 90% of the dose delivered at 24 h. No major differences can be established between these profiles, as confirmed by the *f*_2_ similarity factor. In terms of differences in TFV release according to the medium, a more controlled release of the drug is found in SVF than in the SVF/SSF mixture for both fd1H and fd2H until 48 h. This is corroborated by the *f*_2_ factor, which found no similarity between their profiles in one medium or the other. This could respond to the swelling results, such that the greater swelling of H batches in SVF could translate into greater control over the release of TFV than in the SVF/SSF mixture. 

Based on these results, the batches containing pectin or HPMC are the best suited to the proposed objective, as they allow a controlled release of TFV in SVF that is accelerated in the presence of SSF. It should be highlighted that the fastest TFV delivery in the presence of SSF occurs in the case of fd2P and fd3P, whose profiles are not equivalent to the others according to the *f*_2_ data. This is supported by the high solubility of pectin at a pH equal to or higher than 7, as mentioned earlier. This demonstrates that the pH of the medium influences the behaviour of the pectin formulations, as we stated in the objective of this work. We can therefore confirm that these are pH-responsive systems that can be called “smart”, and would constitute a useful tool for preventing infection in women, since when intercourse takes place with the partner infected by HIV, the release of TFV from the freeze-dried bigel is accelerated in the presence of the seminal fluid—with a pH of around 7.5—thus increasing the ability to fight against the sexual transmission of the virus. 

The main results obtained from fitting the drug release results to Korsmeyer-Peppas and Higuchi kinetics are shown in Table 2. The TFV release profiles of all the batches can be said to have a good fit to both models. 

The drug release profiles of batches containing pectin in SVF have the highest correlation coefficients for the Korsmeyer-Peppas kinetic. The value of the *n* exponent between 0.5 and 1.0 indicates an “anomalous transport” based on drug diffusion and structural modification of the dosage forms as the mechanism responsible for TFV release. The fact that *n* is closer to 0.5 than to 1.0, and the high correlation coefficient for the Higuchi model, point to the major role of the diffusion process in TFV delivery from fd2P and fd3P in this medium. In the SVF/SSF mixture, fd2P and fd3P best fit the Korsmeyer-Peppas kinetic model. *n* values close to and over 1.0 point to an “anomalous transport” and a “transport super-case II”; the main cause of TFV release from these formulations in this medium is their structural changes. This is supported by the values of K_H_ from the Higuchi model, which are significantly higher in the SVF/SSF mixture than in SVF for these batches. The mechanisms responsible for the release of TFV from P batches are reflected in the swelling profiles, which in the SVF/SSF mixture are mostly weight loss by destructuration. This would also justify the slower drug release in SVF, where pectin forms a gel and diffusion and structural modification occur in the dosage form; and the faster delivery in the mixture, where pectin dissolves and there are mainly structural changes in the freeze-dried bigel. 

In batches containing chitosan, fd2C has the highest correlation coefficient for the Higuchi kinetic in both SVF and the SVF/SSF mixture. The mechanism involved in the release of TFV from these freeze-dried bigels is diffusion. However, the profile in SVF also presents a good fit to Korsmeyer-Peppas. Its *n* value is close to but slightly higher than 0.5, showing that both diffusion and structural changes in the formulation induce the release of the drug in this medium. The same occurs for fd3C. This batch best fits the Higuchi model in the SVF/SSF mixture, and diffusion is again the process responsible for the drug release. In SVF, a high correlation coefficient was obtained for Higuchi but even higher for Korsmeyer-Peppas, with a value of *n* between 0.5 and 1.0. This indicates that both diffusion and structural changes cause the release of TFV from fd3C in this medium. The mechanisms explaining the release of TFV from batches containing chitosan can be deduced from the swelling profiles. Their weight decrease in SVF by dissolution of chitosan would explain the structural modification, and the absence of any weight decrease in the systems due to the insolubility of the polymer in the SVF/SSF mixture points to diffusion as the main mechanism of drug release. 

In the case of H batches, the drug release profiles in SVF show a very good fit to the Korsmeyer-Peppas model. Since *n* is lower than 0.5, diffusion can be said to be the only causal mechanism of TFV release from fd1H and fd2H in this medium. The best fit for fd2H is observed for the Higuchi model, thus confirming the above. In the SVF/SSF mixture, diffusion is again the main cause of drug release, as the highest correlation coefficients were obtained for the Higuchi kinetic. This is supported by *n* values from Korsmeyer-Peppas of close to 0.5; fd1H and fd2H also had a good fit to this model in the SVF/SSF mixture. Nevertheless, these values of *n* are slightly higher than 0.5 and thus higher than those obtained for these batches in SVF. This indicates that the structural modification of fd1H and fd2H could play a role in the release of the drug in the SVF/SSF mixture, which could lead to less control over TFV delivery in this medium than in SVF. The release of the drug by diffusion is correlated with the swelling profiles of these batches, which show more sustained losses of structure after the maximum swelling value than in batches containing another polymer. The release profiles can also be explained by these mechanisms. The diffusion of the drug through the gel layer means the delivery of TFV from batches containing HPMC is more controlled in SVF than in those containing pectin or chitosan. 

Regardless of the correlation coefficient values, the results of the statistical analysis by the AIC showed minimum values for Higuchi kinetics in all cases, indicating that diffusion is the mechanism that best explains the release of TFV from the freeze-dried bigels. 

## 4. Conclusions

The nature and proportion of the polymer included in the hydrogel of the freeze-dried bigels based on pectin, chitosan, or HPMC determine the formation and characteristics of the bigel obtained. A system containing 3% *w*/*w* of HPMC does not yield a homogeneous bigel since the high viscosity of this hydrogel hinders its interaction with the organogel; and although bigels can be obtained from hydrogels containing 1% *w*/*w* of pectin or chitosan, freeze-drying produces overly fragile structures (poor mechanical stability) thus impeding their handleability. 

Critical parameters for obtaining optimal dosage forms for the proposed goal (hardness and deformability, mucoadhesion, and pH-dependant drug release) are also conditioned by the nature and/or proportion of the polymer included in the hydrogel of these systems. Hence the batch containing the high proportion of pectin (fd3P) has the best mechanical properties in terms of hardness and resistance to deformation, which would translate into easier handleability and greater suitability for vaginal administration. The presence of pectin in these formulations confers notable mucoadhesive properties, which may be due to the multiple mechanisms involved in its mucoadhesion, and could lead to higher adherence by patients as the formulation would be retained at the site of action. The results of the drug release tests confirm that the batches based on pectin can be called “smart”, since due to the acid character of this polymer, they show a pH-dependent behaviour that is reflected in the swelling profiles and allows a faster release of TFV in the presence of SSF than the other polymers studied. This would give women greater protection against the transmission of HIV from the seminal fluid of an infected partner during sexual intercourse.

## Figures and Tables

**Table 1:** Proportion of the components (% *w*/*w*) in the formulated systems.

Batch	Organogel		Hydrogel	Polymer in the Hydrogel	Tween^®^60
	Sesame Oil	Span^®^60			
1P	37	5.8	55.5	1 (pectin)	1.7
2P	37	5.8	55.5	2 (pectin)	1.7
3P	37	5.8	55.5	3 (pectin)	1.7
1C	37	5.8	55.5	1 (chitosan)	1.7
2C	37	5.8	55.5	2 (chitosan)	1.7
3C	37	5.8	55.5	3 (chitosan)	1.7
1H	37	5.8	55.5	1 (HPMC)	1.7
2H	37	5.8	55.5	2 (HPMC)	1.7
3H	37	5.8	55.5	3 (HPMC)	1.7

**Table 2:** Main parameters resulting from fitting the TFV release profiles to Korsmeyer-Peppas and Higuchi kinetics and their corresponding SSR and AIC values.

Batch (Medium)	Kinetics							
	Korsmeyer-Peppas				Higuchi			
	*r* ^2^	*n*	SSR × 10^2^	AIC	*r* ^2^	*K*_H_	SSR × 10^2^	AIC
fd2P (SVF)	0.9988	0.6243	0.1828	−33.8282	0.9918	0.2623	0.2752	−45.1627
fd3P (SVF)	0.9987	0.6618	0.2843	−37.0410	0.9857	0.2457	0.4250	−41.6863
fd2P (SVF/SSF)	0.9887	1.0020	4.7743	−5.1257	0.9769	0.4360	1.2005	−24.5348
fd3P (SVF/SSF)	0.9990	0.8251	0.0626	−18.1277	0.9814	0.3896	1.0660	−29.7891
fd2C (SVF)	0.9938	0.5563	0.9602	−28.5206	0.9952	0.2451	0.1412	−50.5031
fd3C (SVF)	0.9965	0.6158	0.3830	−23.8249	0.9925	0.2871	0.3030	−44.3930
fd2C (SVF/SSF)	0.9528	0.7640	8.2576	−8.4702	0.9839	0.2990	0.7085	−37.5984
fd3C (SVF/SSF)	0.9731	0.6718	3.5662	−12.6684	0.9838	0.2756	0.6056	−38.8534
fd1H (SVF)	0.9945	0.4693	0.6021	−31.7872	0.9705	0.1558	1.1887	−37.8910
fd2H (SVF)	0.9901	0.4605	1.0454	−27.9251	0.9914	0.2006	0.1686	−49.0818
fd1H (SVF/SSF)	0.9471	0.5522	6.6216	−12.2890	0.9721	0.2614	0.9530	−35.2264
fd2H (SVF/SSF)	0.9885	0.5219	1.2367	−22.3566	0.9963	0.2643	0.1259	−51.4211
